# Evaluation of Comparative Efficacy of Polyherbal Steam Inhalation Versus Polyherbal Nasal Fumigation (Dhoopana) in Children With Rhinitis (Pratishyaya): Protocol for an Open-Label Randomized Controlled Trial

**DOI:** 10.2196/58197

**Published:** 2025-02-21

**Authors:** Monika Kakar, Renu Rathi, Deepthi Balakrishnan, Bharat Rathi

**Affiliations:** 1 Deptartment of Kaumarbhritya Mahatma Gandhi Ayurveda College Hospital & Research Centre Wardha, Maharashtra India; 2 Department of Kaumarbhritya Cheruthuruthy Poomulli Neelakandan Namboodirippad Memorial Ayurveda Medical College Kerala India; 3 Department of Rasa Shastra and Bhaishajya Kalpana Mahatma Gandhi Ayurveda College Hospital and Research Centre Wardha, Maharashtra India

**Keywords:** pratishyaya, Ayurveda, allergic rhinitis, rhinitis, dhoopana, nasal fumigation, steam inhalation, polyherbal, vasa, nirgundi, nilgiri, tulsi, sneezing, nasal cavity, nasal decongestants, evaluation, efficacy, child, adolescent, randomized controlled trial

## Abstract

**Background:**

Rhinitis is a condition characterized by inflammation of the nasal mucosa. It causes obstruction and congestion in the nasal cavity. Clinically, it resembles *pratishyaya* (rhinitis) in Ayurveda, which is caused by accumulation and downward movement of the *tridoshas* (3 elements, named *vata*, *pitta*, and *kapha*) in the nasal cavity. Rhinitis is one of the most common diseases among children. There is no role for antibiotics in rhinitis, and nasal decongestants have also not been found to be effective in its management. In Ayurveda, *dhoopana* (nasal fumigation) is mentioned in the *pratishyaya* treatment protocol. However, we have found no previous study regarding its efficacy. The efficacy of *tulsi*, *vasa*, *nirgundi*, and *nilgiri* is already proven when they are used for steam inhalation in respiratory tract infections. Therefore, in this study, a *dhoopana* of a polyherbal formulation containing *tulsi*, *vasa*, *nirgundi*, and *nilgiri* will be compared with the inhalation of steam containing *arka* (a liquid obtained by distillation) of *tulsi*, *vasa*, *nirgundi*, and *nilgiri* leaves in children with *pratishyaya.*

**Objective:**

We aim to evaluate the efficacy of polyherbal steam inhalation as a standard control against *dhoopana* in children aged 7 to 14 years with *pratishyaya*.

**Methods:**

A total of 70 participants fulfilling the inclusion criteria were selected and distributed into 2 groups of 35 each. The intervention group received *dhoopana* and the control group received polyherbal steam inhalation, both twice daily for 7 days. The primary outcome measure was the change in Total Nasal Symptom Score and a modified cold spatula test. At the same time, the association between *prakriti* (body constitution) and the prevalence of *pratishyaya* in children was analyzed as a secondary outcome. Assessments were performed on days 3, 5, and 7, with a follow-up time of 28 days. Appropriate descriptive and inferential statistics will be used for data analysis.

**Results:**

As of November 2024, we have completed our enrollment of 70 patients, with 35 patients in each group. Data analysis will be completed by February 2025, and we expect results to be published in March 2025.

**Conclusions:**

We anticipate that polyherbal nasal fumigation will be found to be equally as effective as polyherbal steam inhalation in the management of acute rhinitis in the pediatric population. This study may provide a standardized, herbal, safe, and cost-effective treatment for rhinitis in children in the form of *dhoopana*.

**International Registered Report Identifier (IRRID):**

DERR1-10.2196/58197

## Introduction

### Overview

Rhinitis is defined as inflammation of the nasal mucosa due to any infection, allergy, or injury. Symptoms of rhinitis are sneezing, discharge from the nose, obstruction in the nasal cavity, irritation in the nasal cavity, body ache, a feverish sensation, and headache [1]. As per Ayurveda, a clinical picture of rhinitis is seen in the disease *pratishyaya*. It is one of the *nasagata roga* (nasal disorders) in which *kaphadi tridoshas* (the 3 elements of the body) are continuously eliminated through the nose. These vitiated *doshas* accumulate in the head, and their further movement toward the nose causes *pratishyaya* [2]. According to Acharya Charaka, the definition of *pratishyaya* is “*pratikshnam shyayatiitipratishyaya*,” which means the “continuous outward movement of *vata*, *pitta*, and *kapha* doshas from the nostrils.” The prevalence of nonallergic rhinitis is about 40% [3]. The prevalence of allergic rhinitis in India was reported as “11.3% in children aged 6-7 years and 24.4% in children aged 13-14 years” [4]. Acharya Videha explained the clinical presentation of *pratishyaya* as excessive secretions from the nasal cavity and eyes, fever, and generalized weakness with severe headache [2]. *Pratishyaya* is classified into 5 types: *vataja*, *pittaja*, *kaphaja*, *sannipataja*, and *raktaja pratishyaya* [2,5]. The nasal mucosa has a rich blood supply, and stimulation of the sympathetic nervous system causes vasoconstriction that results in shrinkage of the nasal mucosa on the other side, while stimulation of the parasympathetic nervous system is responsible for excessive secretion from the nasal mucosa along with dilatation of local vessels. Emotional disturbance also plays a significant role, as the autonomic nervous system innervation of the nasal mucosa is under the control of the hypothalamus [3]. If no treatment is given at an early stage, the condition may become complicated and lead to comorbid conditions like chronic rhinitis, cough, or breathing difficulty and debility [2]. There is no use of antibiotics in acute rhinitis [6]. Antihistamine decongestants are frequently used in cough and cold. However, in some studies, these were not found to be effective in the management of rhinitis [7]. There are limited data regarding the safety of pseudoephedrine and phenylephrine in rhinitis [8]. In Ayurveda, various oral medications are available for the management of this condition. However, it is difficult to administer Ayurvedic medicines orally to children. So, this study has been planned to find an effective way to manage the acute stage of rhinitis that can be administered locally in the nasal cavity. Various Ayurvedic interventions are mentioned in the Ayurveda classics as protocols for the management of *pratishyaya*, including *dhoopana* (nasal fumigation), an intervention that can effectively manage the disease. In this intervention, various herbs are made into a stick known as a *varti* (wick), and its fumes are used for local fumigation of the affected area. It can be considered an Ayurvedic therapy that delivers the medicines directly into the airways, providing relief and protection to the local region [9].

*Pratishyaya* treatment should be aimed at relieving the *avarodha* (obstruction) created by the dosha. Drugs having *ushna* (hot) and *tikshna* (pungent) properties are indicated in *dhoopana*. The efficacy of steam inhalation of *tulsi*, *vasa*, *nirgundi*, and *nilgiri* has already been shown in respiratory tract infections [10]. So, in this study, *dhoopana* of a polyherbal formulation containing *tulsi*, *vasa*, *nirgundi*, and *nilgiri* will be compared with a steam inhalation containing *arka* of *tulsi*, *vasa*, *nirgundi*, and *nilgiri* in *pratishyaya* in children. *Kashyapa Samhita* (an Ayurveda classical text) is mainly devoted to the Kaumarbhritya branch (Ayurvedic pediatrics) of Ashtanga Ayurveda, and *dhoopana* is described in the treatment protocol of *pratishyaya* [11]. There are many studies regarding the efficacy of oral medication in *pratishyaya* but there is no previous study available regarding the efficacy of local fumigation with medicated fumes in *pratishyaya* in children. Steam inhalation has a known soothing effect on the nasal mucosa in rhinitis. Herbal steam inhalation is more effective as compared to plain water steam inhalation [12], but steam inhalation is quite difficult to administer in children. So, in this study, we plan to compare the effect of polyherbal steam inhalation with polyherbal fumigation in children with *pratishyaya*. The herbs chosen for *dhoopana* were justified in previous studies, as steam inhalation of *tulsi*, *vasa*, *nirgundi*, and *nilgiri* was found to be effective for reducing local inflammation in the nostrils. A detailed description of previous articles and a research gap analysis with justification for this study is given in Table 1.

**Table 1:** Previous studies and research gap analysis.

Reference	Conclusion	Remark
Memon [13]	*Vyoshadi vati* was found to be effective in reducing signs and symptoms of *pratishyaya* (rhinitis) in comparison with chlorpheniramine maleate in children aged 4-8 years.	Children tend to resist the intake of medicines orally in most cases, especially when they are sick. So, local fumes or steam inhalation could be a better choice.
Tarun and Anup [14]	*Nasya* with *tulasi swarasadi taila* and *haridrakhand* showed 100% results in objective as well as subjective parameters of *pratishyaya*. *Tulasi swarasadi taila nasya* and *haridrakhand* gave effective results in treating *pratishyaya*.	As *tulsi* has an anti-inflammatory effect and was found to be effective in the management of *pratishyaya*, and the *nasya* procedure is contraindicated in the acute stage, fume inhalation can be done with the use of *tulsi* in place of *nasya karma.* Local fume inhalation can provide instant relief for the symptoms of *pratishyaya*.
Rajput and Patni [15]	According to Acharya Charaka, any drug when instilled into the nostrils causes direct action in the brain; hence, it is highly effective in the treatment of various diseases related to the nervous system and supraclavicular region. The *nasya* procedure is indicated in *pratishyaya*. Standardization of *nasyakarma* with Ayurveda and modern scientific parameters with proper documentation is a current need.	*Nasya* is widely used in rhinitis but it is contraindicated in its acute stage. Moreover, it is quite difficult to administer to children. Direct contact with oil in the nostrils sometimes irritates children. In this study, acute cases were mentioned in the inclusion criteria, so local fumes could provide a better approach to the treatment of *pratishyaya*. Moreover, local nasal fumigation is described in the treatment protocol of *pratishyaya*, but no previous study has been done to determine its efficacy in rhinitis.
Kamble et al [16]	Steam inhalation of *tulsi* leaves was found to be more effective in reducing sign and symptoms of cough and cold in adults than steam inhalation of plain water.	*Tulsi* steam inhalation was found to be better than plain steam inhalation in the adult population. This shows that volatile herbs like *tulsi* have better efficacy when given locally in the form of vapors. Steam inhalation is also easier to administer in children. So in this study, *tulsi* was chosen as one of the herbs for fume as well as steam inhalation.
Kumar Dwibedi et al [17]	Initially, there was mild relief in symptoms. Gradually, improvement was seen in signs and symptoms at further appointments. In the last appointment, the patient felt relief and could perceive smells.	Atrophic rhinitis is a chronic stage of *pratishyaya*; hence, a combination of *nasya* and *dhoompana* was given to the patient. *Trikatu dhoompana* was found to be effective in the management of the disease in the chronic stage. Thus, *dhoopana* (nasal fumigation) should only be given to children in the acute stage of *pratishyaya*.
Gowrishankar et al [10]	Phytochemicals from selected plants were found to be effective against protein targets of COVID-19.	In an in silico study, the active components of *vasa*, *nirgundi*, and *nilgiri* were found to be effective against protein targets of SARS‑CoV‑2. Hence, these herbs were chosen for the intervention as well as the control group.
Swain and Sahu [18]	There was improvement in symptoms of COVID-19 after taking steam inhalation. The severity and duration of infection was reduced after steam inhalation. It was found to be a ray of hope in that pandemic.	This was a nonrandomized, nonclinical trial. The study was performed along with oral medications for COVID-19 without confirming the efficacy of steam inhalation alone. So there is a need for a study of herbs as a single therapy in children. This study is a randomized clinical trial with the use of polyherbal steam inhalation in the control group of children without oral medication.
Berger et al [19]	The intranasal formulation of azelastin and fluticasone propionate was found to be safe for administration in children, and it was well tolerated for 3 months by children with allergic rhinitis.	Epistaxis was reported as a treatment-related adverse effect in both groups, followed by headache and other adverse effects. Herbal procedures are quite safe and effective. Local polyherbal fume administration can be an alternative.
Sebastian and Sujatha [20]	The study showed that steam inhalation with *tulsi* leaves is effective in relieving symptoms of coryza compared to hot water steam inhalation in children aged 6-12 years.	The study was performed in a selected community with a small sample size, and no randomization was done. Steam inhalation was administered for only 3 days. The follow-up time was not mentioned in the article. This study is a randomized clinical trial, and *tulsi* along with *nilgiri*, *nirgundi* and *vasa* will be used for steam inhalation in the control group for 7 days with a follow-up of 28 days.
Macchi et al [21]	A statistically significant improvement was seen in ciliary motility in the first group, who received a nasal wash with sodium hyaluronate in saline solution (*P*<.001).	This was a pilot study with very small sample size. Moreover, the study included patients with recurrent upper respiratory tract infections; there was no specific disease chosen for the study. Aerosol use was indicated in lower respiratory tract infection, as there was a chance of the medicine entering the lungs. In our study, only *pratishyaya* cases will be included, rather than all patients with respiratory tract infections. Polyherbal fumes will be administered in the trial group, which could be a better alternative to aerosols.
Baartmans et al [22]	Annually, on average 3 people are admitted to a Dutch burn center for burns resulting from steam inhalation therapy. Most patients are children, and they need skin grafting more often than adults. At burn centers in the Netherlands, 31 patients were admitted with burns caused by steam inhalation therapy in the 1998 to 2007 period.	These data show that steam inhalation can result in burns, especially in children. So, in our study, we plan to find if *dhoopana* can be an alternative.
Murphy et al [23]	Between July 1 and December 31, 2002, 7 children were admitted to the burn unit of a children’s hospital in Dublin with scalds sustained during the course of steam inhalation. The children ranged in age from 9 months to 10 years. Techniques involving kettles or bowls of boiling water should be actively discouraged.	The common technique of steam inhalation is risky in children as it may cause burns. So, in our study, we plan to find an easier way to administer herbal effects to the nasal mucosa through *dhoopana*.

### Aim

We aim to study the efficacy of polyherbal steam inhalation versus *dhoopana* in children with *pratishyaya*.

### Primary Objectives

There are 3 primary objectives: first, to determine the efficacy of *dhoopana* in reducing signs and symptoms of *pratishyaya* compared to polyherbal steam inhalation; second, to study the duration of alleviation of Total Nasal Symptom Score (TNSS); and third, to determine the efficacy of *dhoopana* in the reduction of nasal obstruction in a modified cold spatula test.

### Secondary Objectives

There are 2 secondary objectives: first, to study the prevalence of *pratishyaya*, its origin, and its causative factors, such as viruses, bacteria, or allergies; second, to analyze the association between *prakriti* (body constitution) and the prevalence of *pratishyaya* in children.

### Hypothesis

The alternate hypothesis is that *dhoopana* has equivalent efficacy to polyherbal steam inhalation in reducing the signs and symptoms of *pratishyaya* in children aged 7-14 years. The null hypothesis is that *dhoopana* does not have equivalent efficacy as polyherbal steam inhalation in reducing the signs and symptoms of *pratishyaya* in children aged 7-14 years.

### Trial Design

This will be a randomized, reference, controlled, open-label equivalence clinical study. A total of 70 patients have been recruited for the study.

## Methods

### Recruitment

The patients were selected from the outpatient and inpatient departments of the Department of Kaumarabhritya (Ayurvedic pediatrics) of Mahatma Gandhi Ayurveda College, Hospital & Research Centre and from the surrounding region. After initial screening, the patients were randomized using computer-generated random numbers into 2 groups; the control group (polyherbal steam inhalation) and the intervention group (polyherbal nasal fumigation). All baseline parameters were recorded at the start of the study. Both groups underwent treatment for 7 days. All the parameters were recorded on the third, fifth, and seventh days, with a follow-up of 28 days after the treatment period. Patients were advised to drink warm water and stay away from cold and sour items. Patients were given regular counseling and reminders through calls or messages to follow the rules and regulations of the trial.

### Guidelines

**Figure 1:**
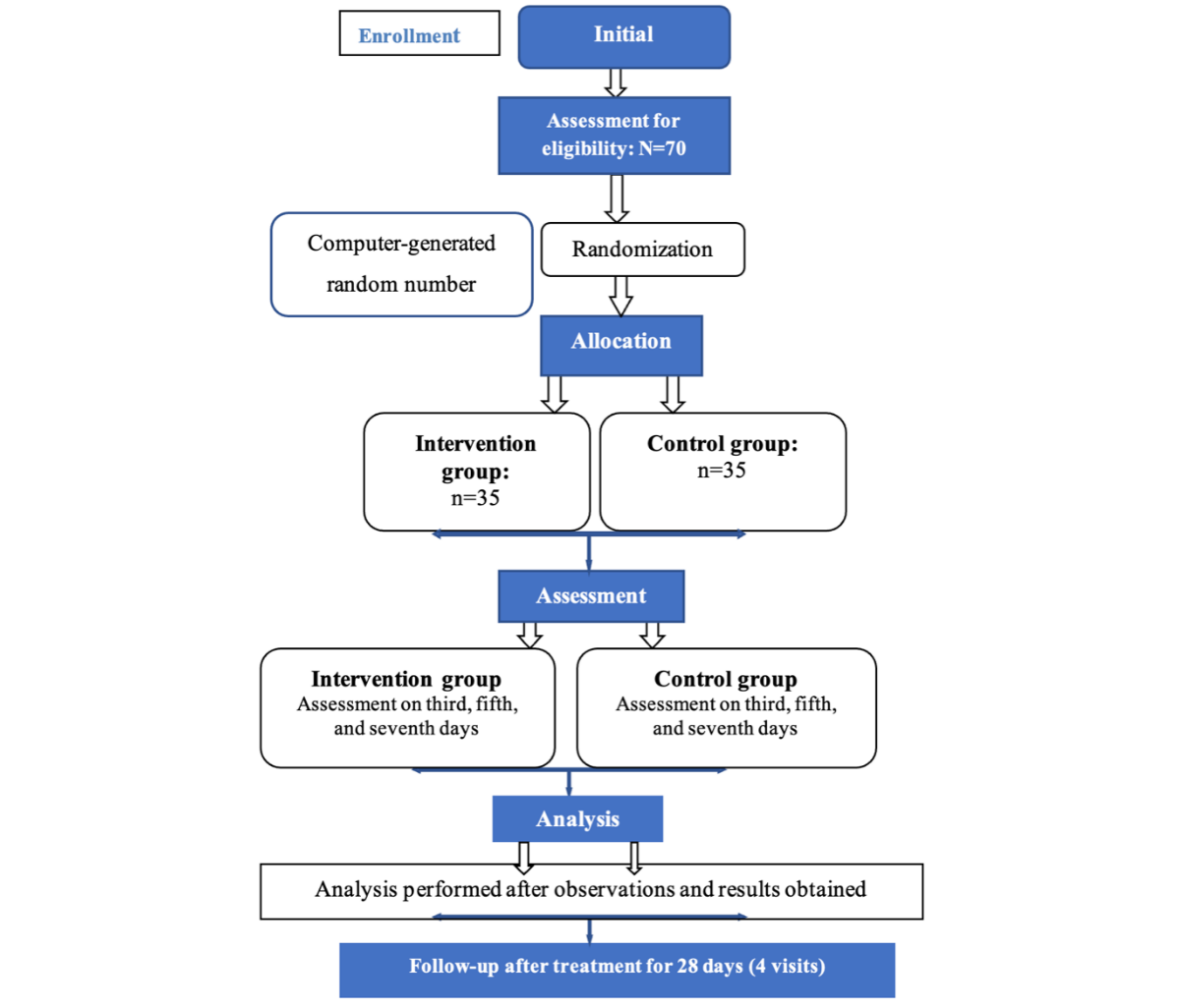
Consolidated Standards of Reporting Trials (CONSORT) flow diagram.

This study used the Consolidated Standards of Reporting Trials (CONSORT) guidelines; Figure 1 shows a flow diagram of the study.

### Grouping and Dosage

Grouping and dosage are described in Table 2.

**Table 2:** Grouping and dosage.

	Control group (polyherbal steam inhalation)	Intervention group (*dhoopana*; polyherbal nasal fumigation)
Participants, n	35	35
Name and details of the medication	*Arka* is prepared from *tulsi*, *vasa*, *nirgundi*, and *nilgiri*. In participants with mild symptoms, 2.5 ml *arka* is added to 500 ml of water, and in those with moderate to severe symptoms, 5 ml *arka* is added to 500 ml of water for steam inhalation.	*Dhoomvarti* (polyherbal wick) prepared from dry leaves of *tulsi*, *vasa*, *nirgundi*, and *nilgiri*.
Mechanism of delivery	Steam	Fumes
Duration	1 minute for mild symptoms and 1.5 minutes for moderate to severe symptoms.	1 minute for mild symptoms and 1.5 minutes for moderate to severe symptoms.
Route/mode of administration	Nostrils	Nostrils
Frequency	Twice daily	Twice daily

### Inclusion Criteria

Children of either sex aged between 7 to 14 years whose parents provide written consent for enrolling their child in the study will be included if they have had the common cold with features of *pratishyaya* for 7-10 days.

### Exclusion Criteria

Patients will be excluded if they have had the common cold for more than 10 days or if they have chronic allergic rhinitis, *dushta pratishyaya* (chronic rhinitis with complications), *raktaja pratishyaya* (a type of rhinitis with dominance of the blood element), *sannipataja pratishyaya* (rhinitis with involvement of all 3 elements), or infectious diseases such as tuberculosis. Patients will also be excluded if they have a cleft palate, a deviated nasal septum, or nasal polyps, or if they are hypersensitive to the trial drug or any of its ingredients.

### Drug Collection and Authentication

The raw material for the drug was purchased from a reliable source and authenticated and identified by the Department of Dravyaguna and Rasashastra. Details of the raw ingredients are provided in Table 3 and images are shown in Multimedia Appendix 1.

**Figure 2:**
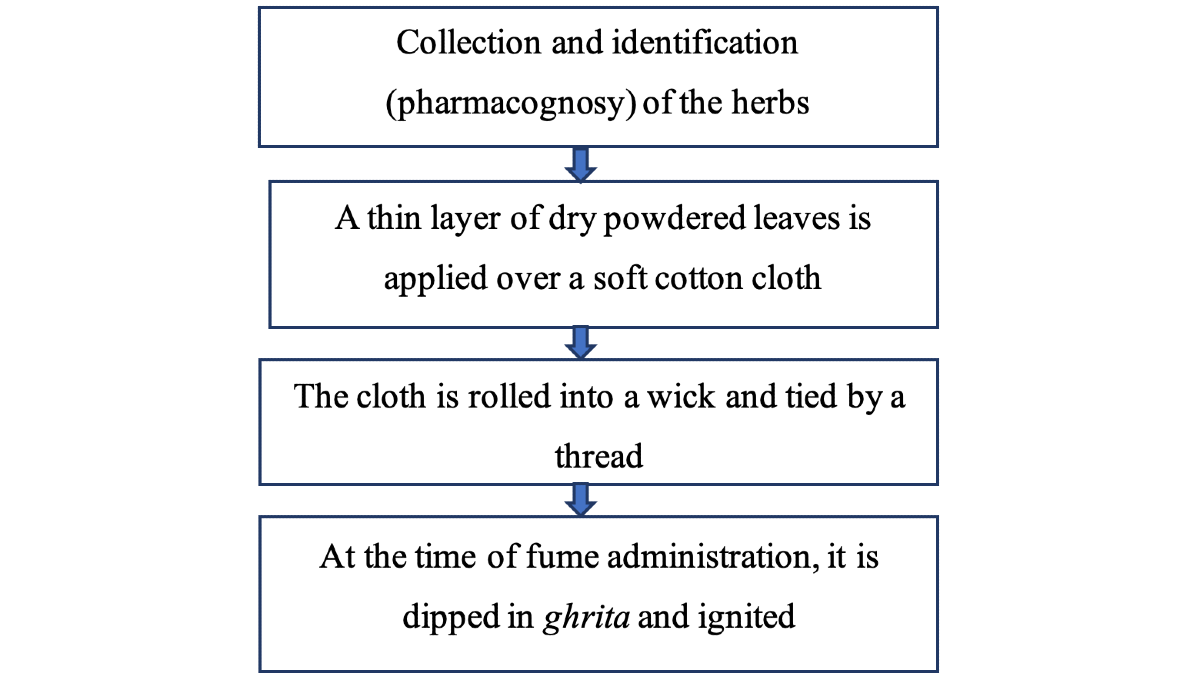
Method of preparation of dhoomvarti.

The *dhoomvarti* (polyherbal wick) for nasal fumigation and *arka* (for steam inhalation) from *tulsi*, *vasa*, *nirgundi*, and *nilgiri* leaves were prepared as per the classical methods and standard protocol at the Mahatma Gandhi Ayurveda College and Hospital and were analyzed in a pharmaceutical laboratory. The method of drug preparation is described in Figure 2.

*Arka* of the leaves of *tulsi*, *vasa*, *nirgundi*, and *nilgiri* was prepared by using a distillation process. Parents of the patients were counseled and trained regarding the administration of *dhoopana* and steam inhalation at their homes.

**Table 3:** Latin name and family name of the herbs used for nasal fumigation and steam inhalation

Serial No.	Drug	Latin name	Family
1	*Tulsi*	*Ocimum sanctum* Linn	Lamiaceae
6	*Nirgundi*	*Vitex negundo* Linn	Verbenaceae
7	*Vasa*	*Adhatoda vasica* Nees	Acanthaceae
8	*Nilgiri*	*Eucalyptus globules* Labill	Myrtaceae

### Outcomes

The outcomes are designed to compare the efficacy of *dhoopana* and polyherbal steam inhalation on *pratishyaya* with subjective and objective parameters.

#### Subjective Criteria

Signs and symptoms of *pratishyaya* will be evaluated with the TNSS, illustrated in Table 4.

**Table 4:** Total Nasal Symptom Score (TNSS).

Symptom and domain			Score
**Symptoms**			
	**Rhinorrhoea**		
		No symptoms	0
		Aware but not troubled (mild)	1
		Troublesome but does not interfere with normal day-to-day activities or sleeping habits (moderate)	2
		Interferes with normal day-to-day activities or sleeping habits (severe)	3
	**Nasal itching**		
		No symptoms	0
		Aware but not troubled (mild)	1
		Troublesome but does not interfere with normal day-to-day activities or sleeping habits (moderate)	2
		Interferes with normal day-to-day activities or sleeping habits (severe)	3
	**Nasal obstruction**		
		No symptoms	0
		Aware but not troubled (mild)	1
		Troublesome but does not interfere with normal day-to-day activities or sleeping habits (moderate)	2
		Interferes with normal day-to-day activities or sleeping habits (severe)	3
	**Sneezing**		
		No symptoms	0
		Aware but not troubled (mild)	1
		Troublesome but does not interfere with normal day-to-day activities or sleeping habits (moderate)	2
		Interferes with normal day-to-day activities or sleeping habits (severe)	3
**Additional symptoms as per Ayurveda texts**			
	***Sirashoola*** **(headache)**		
		No headache	0
		Occasional headache	1
		Frequent headache	2
		Continuous headache	3
	***Aruchi*** **(loss of appetite)**		
		Absent	0
		Present	1
	***Kasa*** **(cough)**		
		No cough	0
		Occasional cough	1
		Moderate cough	2
		Continuous cough with throat and chest pain	3

#### Objective Criteria

The modified cold spatula test will be used to measure nasal patency in rhinitis. The test is done to study the area of fogging associated with a nasal obstruction and compare the pre- and posttreatment outcomes. The patients are asked to breathe normally over a polished stainless steel plate, and the extent of fogging is marked with a marker pen. The magnitude of fogging is then measured with a transparent graph sheet marked with a central red line at 50 mm on the x-axis, dividing the graph into 2 equal parts with corresponding right and left areas of fogging.

### Sample Size

A comparative pilot study was performed with 20 patients, including 10 patients in 2 groups, to evaluate the efficacy of *dhoopana* for rhinitis in comparison with polyherbal steam inhalation. Based on the results of that study, the minimum sample size for this study was calculated by using the following formula of proportion:




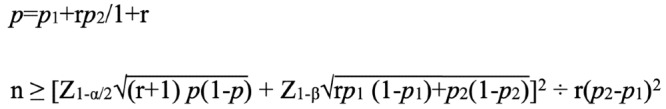




The parameters related to sample size were as follows: α=.05; β=.2; proportion in group 1=0.60; proportion in group 2=0.90; ratio (group 2/group 1)=1; minimum sample size needed for group 1=32; and minimum sample size needed for group 2=32. The minimum total sample size needed was 64, and the minimum total number of patients to be enrolled was 35 in each group (including a 10% withdrawal rate).

### Data Collection Tools

This study will use the *Ayurveda Samhitas* (a textbook on Ayurveda), modern texts, and an online search of PubMed and Google Scholar. The polyherbal *dhoomvarti* will be prepared from a powder of *tulsi*, *vasa*, *nirgundi*, and *nilgiri*. The polyherbal *arka* will be prepared from *tulsi*, *vasa*, *nirgundi*, and *nilgiri*. The study will also use a case record form, patient information sheet, and written informed consent form.

### Data Analysis

Data will be analyzed by using appropriate descriptive and inferential statistics. Quantitative variables will be analyzed with the Student *t* test and subjective parameters will be analyzed with the Wilcoxon signed test and Mann-Whitney *U* test. Alternate statistical methodologies may be applied if deemed necessary.

### Ethical Considerations

The study obtained approval from the institutional ethics committee of Mahatma Gandhi Ayurved College Hospital and Research Centre (MGACHRC/IEC/JULY-2022/522). The committee will decide on the end point and oversee the trial as it progresses. The researchers will assess any adverse events and will report to the ethics committee. Consent from parents and assent from patients was obtained before conducting the trial in the local language while explaining every aspect of the study. The informed consent form included a patient information sheet (part 1) and a certificate of consent (part 2). Data will be anonymized and personal information of the participants will be kept confidential before, during, and after the trial. Physical data will be stored in a protected storage facility with access available only to the researchers. Computerized data will be held in a password-protected hard drive with access available only to the researchers. There was no need to provide any compensation to the patients in the trial. Identification of individual participants and users will not be possible in any images in the manuscript or supplementary files. If needed, prior consent will be obtained from identifiable individuals before the inclusion of such images.

### Withdrawal Criteria and Stoppage

No adverse effects of the trial drug were noted during the treatment period. Thus, no cases were excluded from the study. A total of 4 patients, including 2 patients in each group, voluntarily stopped taking treatment during the trial and were counted as withdrawn.

### Dissemination

This protocol will be published and disseminated as a thesis on *pratishyaya*. The study protocol provides a detailed overview of the study design, methodology, data collection procedures, data analysis plan, and ethical considerations. By disseminating this protocol, we hope to advance knowledge in the field and facilitate future research.

## Results

As of November 2024, we completed the enrollment of 70 patients, with 35 patients in each group. A total of 4 patients voluntarily withdrew from the trial. Data analysis of 66 patients will be completed by February 2025, and we expect the results to be published in March 2025.

## Discussion

*Dhoopana* is mentioned in the Ayurveda classics for the management of acute rhinitis, and we anticipate that it will be found to be equally effective as polyherbal steam inhalation in the management of acute rhinitis in children. Steam inhalation of the herbs that were chosen for this study has been found to be effective in the management of upper respiratory tract infections. Thus, fumes of the same ignited herbs (ie, *tulsi*, *vasa*, *nirgundi*, and *nilgiri*) should work just as well in the management of rhinitis. The disease *pratishyaya* is very common in the pediatric population, but no standardized Ayurvedic therapy is available that can be given locally in the nostrils in its acute stage. Locally given medicines are fast and effective compared to oral medication. Another classical procedure known as *nasya* (nasal instillation of oil) is recommended for rhinitis, and as per previous research, it is effective in the management of chronic and recurrent rhinitis, but as per the Ayurveda classics, it cannot be used in acute rhinitis. The herbs chosen for this study are hot and pungent in nature and can reach the minute *srotasa* (channels of the nostrils); thus, they promise to be beneficial in acute rhinitis. Polyherbal *dhoopana* is safe and easy to administer, and if it is found to be effective, it may be a better alternative to steam inhalation. Thus, it can be used in general pediatric practice for patients with acute rhinitis. A positive outcome of the study will facilitate further studies in a large population with standardization of the procedure for *dhoopana*. In this study, a polyherbal wick was prepared manually, and it takes some time to ignite the herbal powder. In the future, this can be improved by using a pocket-friendly herbal nasal fumigator that ignites the herbs in few seconds without any manual practice. This could be patented and copyrighted later and publicized for the benefit of the population.

## Appendix

Multimedia Appendix 1 Images of intervention.

## Abbreviations

CONSORT: Consolidated Standards of Reporting Trials
TNSS: Total Nasal Symptom Score

## Data Availability

The data sets generated during and/or analyzed during this study are available from the corresponding author upon reasonable request.
