# Supplementary material for: Evaluation of Comparative Efficacy of Polyherbal Steam Inhalation Versus Polyherbal Nasal Fumigation (Dhoopana) in Children With Rhinitis (Pratishyaya): Protocol for an Open-Label Randomized Controlled Trial
Source: JMIR Res Protoc. 2025 Feb 21;14:e58197. doi: 10.2196/58197 (PMC11890129; doi:10.2196/58197)
Supplement: Multimedia Appendix 1 [file resprot_v14i1e58197_app1.docx]

## Appendices:


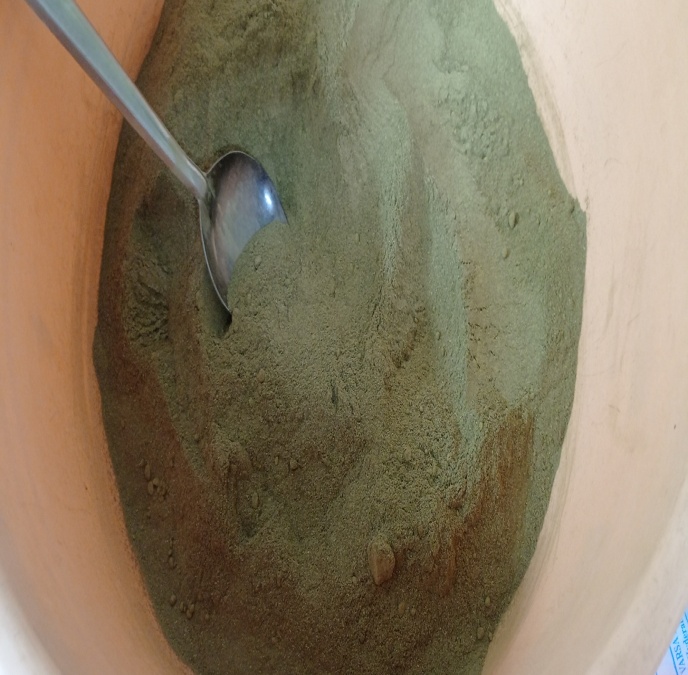

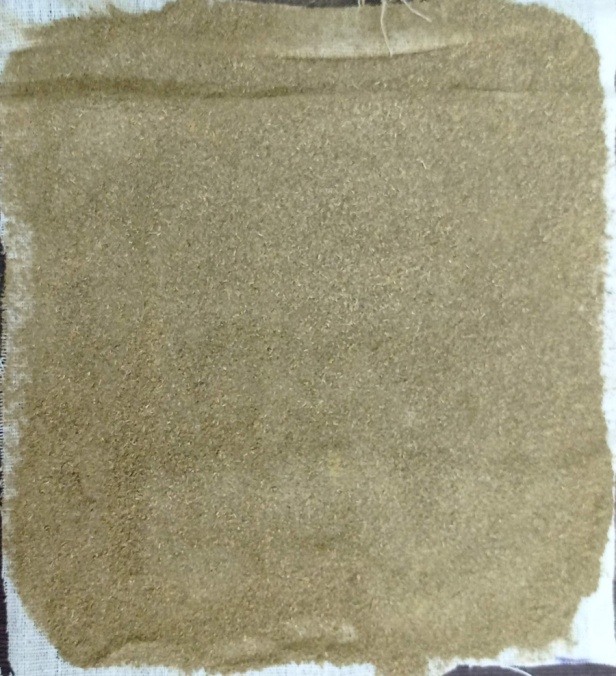


**Fig 1. Powder of the herbs used for the preparation of *Dhoomvarti* (polyherbal wick)**


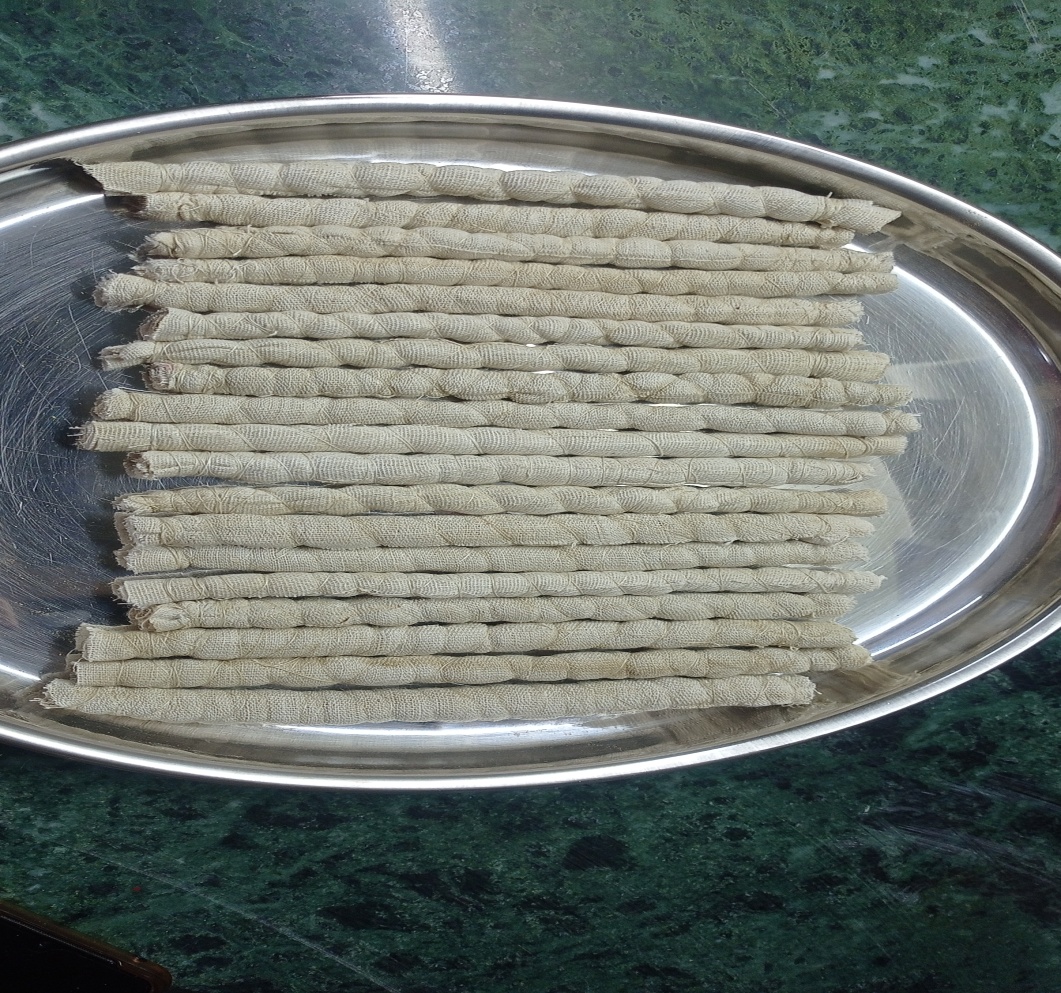


**Fig 2. Prepared *Dhoomvartis* (Polyherbal wicks) for nasal fumigation**

**
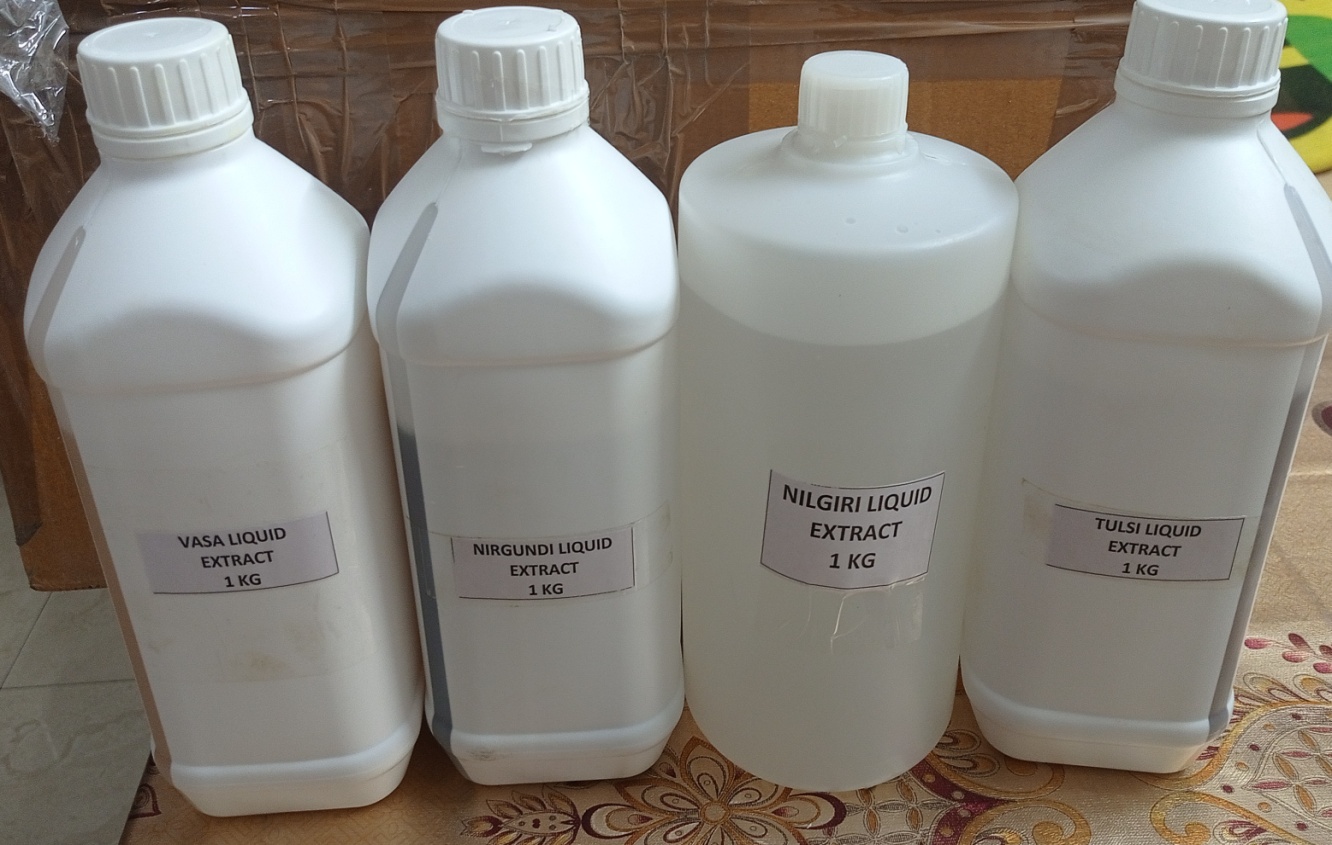
**

**Fig 3. Prepared liquid extracts for steam inhalation**
